# Impact of Early Allograft Dysfunction on Renal Function Outcomes Following Liver Transplantation

**DOI:** 10.3390/medicina62040657

**Published:** 2026-03-30

**Authors:** Jungho Shin, Sanggyun Suh, Suk-Won Suh

**Affiliations:** 1 Department of Internal Medicine, Chung-Ang University Hospital, 102 Heukseok-ro, Dongjak-gu, Seoul 06973, Republic of Korea; junghoshin@cau.ac.kr; 2 Department of Surgery, Chung-Ang University Gwangmyeong Hospital, Gwangmyeong-si 14353, Gyeonggi-do, Republic of Korea; seo861214@cauhs.or.kr; 3 Department of Surgery, Chung-Ang University Hospital, Chung-Ang University College of Medicine, 102 Heukseok-ro, Dongjak-gu, Seoul 06973, Republic of Korea

**Keywords:** liver transplantation, early allograft dysfunction, acute kidney injury, glomerular filtration rate

## Abstract

*Background and Objectives*: Early allograft dysfunction (EAD) after liver transplantation (LT) is associated with adverse graft and patient outcomes. However, its effects on kidney outcomes remain unclear. We retrospectively investigated the association between EAD and various kidney events following LT. *Materials and Methods*: We included 92 LT recipients. EAD was defined by the presence of ≥1 of the following: total bilirubin level ≥ 10 mg/dL or international normalized ratio ≥ 1.6 on postoperative day 7, or aspartate aminotransferase or alanine aminotransferase level > 2000 U/L within the first 7 days post-LT. Kidney outcomes included acute kidney injury (AKI), acute kidney disease (AKD), kidney replacement therapy (KRT) performance, and changes in estimated glomerular filtration rate (eGFR). *Results*: AKI incidence was comparable between the non-EAD and EAD groups; however, stage 3 AKI incidence was higher in the EAD group (65.0% vs. 22.2%; *p* = 0.001). AKD occurred more frequently in the EAD group (75.0% vs. 30.6%, *p* = 0.001). KRT was required more frequently within 7 days of LT in the EAD group (70.0%) than in the non-EAD group (15.3%) (*p* < 0.001). Multivariate analysis identified EAD as an independent predictor of KRT requirement (*p* < 0.001). EAD was associated with prolonged (≥7 days) KRT requirement (*p* = 0.025). In the receiver operating characteristic curves, all EAD components were associated with KRT requirement following LT. The baseline and 3-month eGFR levels were comparable; however, a trend toward a steeper decline was noted in eGFR in the EAD group than that in the non-EAD group (*p* = 0.090). The burden of hospitalization and calcineurin inhibitor exposure were similar between the groups. *Conclusions*: EAD appears to be independently associated with adverse kidney outcomes following LT. Preventive strategies targeting EAD are required to improve post-transplant prognosis and mitigate kidney-related complications.

## 1. Introduction

Liver transplantation (LT) is a life-saving treatment for patients with end-stage liver disease or liver malignancies. Despite substantial advances in surgical techniques, immunosuppression, and perioperative care, several challenges affect graft function and patient outcomes. Early allograft dysfunction (EAD) is a frequent and critical complication following LT, resulting in adverse graft and patient prognoses [1,2]. With a reported incidence of 15–30%, EAD occurrence has been increasing in both deceased and living donor LT, largely due to the increasing use of marginal grafts and the expansion of transplant indications to high-risk recipients [3]. Therefore, identifying the risk factors for EAD and understanding its clinical impact are essential for optimizing the management of LT recipients.

In the management of LT recipients, the kidneys are frequently affected. Post-LT kidney dysfunction, either acute kidney injury (AKI) or chronic kidney disease (CKD), is a common complication arising from multifactorial causes and is associated with poor prognosis and substantial healthcare burden [4,5,6,7,8,9,10]. Thus, preserving kidney function is essential for optimizing patient outcomes after LT. A previous study revealed that EAD is a risk factor for both short- and long-term kidney impairment [11]. However, beyond hard outcomes such as AKI requiring kidney replacement therapy (KRT) or end-stage kidney disease, further comprehensive analysis is needed, including the severity and duration of kidney injury, long-term kidney function trajectories, and the underlying conditions driving kidney function deterioration. The association between EAD and kidney outcomes in living-donor LT recipients remains unclear.

We investigated the effect of EAD on a spectrum of kidney outcomes following LT, including AKI, acute kidney disease (AKD), and CKD. We also evaluated the post-LT morbidities that could influence kidney function, such as hospitalization and exposure to calcineurin inhibitors. By better understanding this relationship, we attempted to identify the connection between EAD and kidney outcomes to inform strategies for mitigating complications after LT.

## 2. Materials and Methods

### 2.1. Patients

This retrospective study recruited a total of 94 adults (≥18 years) who underwent LT at two university medical centers between July 2015 and August 2024. Among the eligible recipients, two patients who underwent retransplantation for graft failure were excluded. No recipients received maintenance KRT before LT. Ultimately, 92 recipients were included and followed up until loss to follow-up, graft failure, death, or study endpoint (September 2024). They received grafts from living donors (61 [66.3%]) and deceased donors (31 [33.7%]). All deceased donors were donation after brain death donors. This study was conducted in accordance with the Declaration of Helsinki and approved by the Institutional Review Board of Chung-Ang University Hospital (Number: 2508-003-19587) on 13 August 2025. The requirement for written informed consent was waived due to the use of a retrospective design and anonymized data.

### 2.2. Data Collection

Baseline demographic and clinical data of recipients and donors were obtained from electronic medical records and included age, sex, body mass index, comorbidities, causes of liver disease, donor type, ABO incompatibility, donor age and sex, cold ischemia time, warm ischemia time, graft steatosis, graft–recipient weight ratio, and perioperative KRT use. Operative factors, including operative time, estimated blood loss, and transfusion volume, were also collected. Pre-LT laboratory values included serum albumin, total bilirubin, aspartate aminotransferase (AST), alanine aminotransferase (ALT), alkaline phosphatase, sodium, and creatinine levels, platelet count, and international normalized ratio (INR).

Baseline kidney function was determined using the nadir serum creatinine within 12 months prior to LT. In cases without available measurements (n = 4), the baseline creatinine level was imputed by back-calculation assuming an estimated glomerular filtration rate (eGFR) of 75 mL/min/1.73 m^2^ [12].

Post-LT data were collected to determine EAD and kidney events. Total bilirubin, AST, and ALT levels and INR within 7 days after LT were measured to define EAD. Data on serum creatinine levels and KRT use were collected during the study period to identify kidney events. Consistent with the Model for End-Stage Liver Disease (MELD) scoring system [13], creatinine levels > 4.0 mg/dL were truncated to 4.0 mg/dL, and recipients receiving KRT were assigned a creatinine value of 4.0 mg/dL. Our institution employs a renal-sparing protocol. If serum creatinine level exceeds the upper normal limit after the first month following LT, tacrolimus exposure is minimized and everolimus is introduced. In addition, these patients are co-managed with a nephrologist during follow-up.

Furthermore, hospitalization events and calcineurin inhibitor exposure after LT were reviewed to assess post-LT morbidity and its potential contributions to kidney outcomes. The severity of hospitalization within 1 year after LT was evaluated in detail. All recipients received tacrolimus; therefore, tacrolimus trough levels were obtained within 1 year of LT.

### 2.3. Definitions

EAD was defined as the presence of one or more of the following criteria (1): total bilirubin > 10 mg/dL on postoperative day 7, INR > 1.6 on postoperative day 7, and AST or ALT > 2000 U/L within the first 7 postoperative days. If the recipients died within 7 days, the last measurements of total bilirubin and INR were used.

AKI and AKD were defined and staged according to the Kidney Disease: Improving Global Outcomes clinical practice guidelines, the joint consensus report of the Acute Disease Quality Initiative, and the Perioperative Quality Initiative [14,15]. AKI was defined by an increase in the serum creatinine level by ≥0.3 mg/dL within 48 h or by ≥1.5 times within the prior 7 days, based on the baseline creatinine. AKI stages were defined as follows: stage 1, an increase in serum creatinine to 1.5–1.9 times of the baseline or by ≥0.3 mg/dL; stage 2, an increase in serum creatinine to 2.0–2.9 times of the baseline; and stage 3, an increase in serum creatinine to ≥3.0 times of the baseline, an increase to ≥4.0 mg/dL, or initiation of KRT. AKD was defined as meeting the criteria for AKI beyond postoperative day 7 after LT.

### 2.4. Outcomes

We first evaluated the incidence of AKI and AKD according to the EAD status. AKI was further categorized as early (≤2 postoperative days) or delayed (>2 postoperative days). Next, we assessed the association between EAD and the composite outcome of KRT requirement within 7 days after LT, and we evaluated the predictive performance of each EAD component for KRT requirement. We then examined whether EAD was associated with prolonged (≥7 days) KRT use. Finally, we compared the baseline and 3-month eGFR values and the eGFR slope after 3 months between the EAD groups. eGFR was estimated using the Chronic Kidney Disease Epidemiology Collaboration equation [16].

Hospitalization events and calcineurin inhibitor exposure were compared between the EAD groups to assess the factors potentially influencing long-term kidney function. Hospitalization rates were compared between the EAD groups. Hospitalization within 1 year after LT was graded using the Clavien–Dindo classification system [17]. On the other hand, tacrolimus exposure was summarized using normalized individual area-under the curve (AUC) measures derived from trough levels over the follow-up period.

### 2.5. Statistical Analysis

Continuous variables are presented as medians (25th and 75th percentiles) and categorical variables as counts (percentages). Between-group comparisons were performed using the Wilcoxon rank-sum test for continuous variables and the chi-squared test for categorical variables. Odds ratios (ORs) for KRT requirement were estimated using logistic regression; the multivariate model included variables with *p* < 0.1 in the univariate analysis and was additionally fitted using Firth’s penalized likelihood method due to sparse data and potential separation. The receiver operating characteristic (ROC) curve for KRT probability was assessed using each EAD criterion, and the AUC was calculated. The eGFR slopes according to EAD status were evaluated using a linear mixed-effects model. Overall patient survival was additionally evaluated according to EAD status using Kaplan–Meier analysis and compared with the log-rank test. Hospitalization rates were analyzed using negative binomial regression with a log link and an offset term, considering the varied follow-up durations across participants. All statistical analyses were conducted using the R software (version 4.4.2; R Foundation for Statistical Computing, Vienna, Austria). Statistical significance was defined as a two-sided *p*-value < 0.05.

## 3. Results

### 3.1. Characteristics in the Non-EAD and EAD Groups

EAD occurred in 20 (21.7%) recipients following LT. Table 1 shows the characteristics of the recipients with and without EAD. Age, sex, comorbid diseases, and baseline eGFR were comparable; however, the cause of liver disease varied. MELD score was higher and KRT was more frequently required before LT in those with EAD than without EAD. In terms of donor factors, recipients with EAD had older donors and a prolonged cold ischemia time.

### 3.2. Post-LT AKI and AKD According to EAD Status

**Figure 1:**
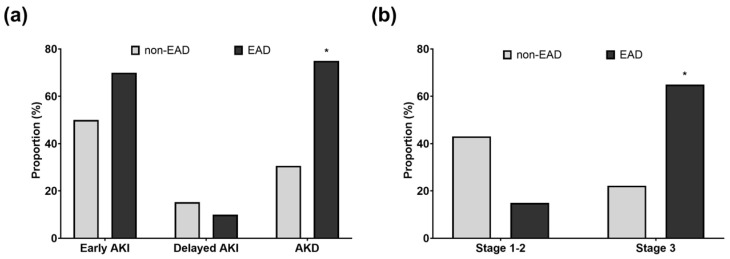
Incidence and timing of AKI and AKD following LT according to EAD status. (**a**) Early AKI (≤2 days) occurred in 50.0% of non-EAD recipients and in 70.0% of EAD recipients (*p* = 0.182), whereas delayed AKI (>2 days) occurred in 15.3% and 10.0%, respectively (*p* = 0.813). AKD was more frequent in the EAD group (75.0% versus 30.6%; *p* = 0.001). (**b**) AKI severity differed between the groups (*p* = 0.001): AKI stage 1–2 occurred in 43.1% and 15.0% in the non-EAD and EAD groups, whereas AKI stage 3 occurred in 22.2% and 65.0%, respectively. * reflects *p* < 0.05 for the comparison between the non-EAD and EAD groups. AKI, acute kidney injury; AKD, acute kidney disease; EAD, early allograft dysfunction.

There were 47 (65.3%) and 16 (80.0%) patients in the non-EAD and EAD groups, respectively (*p* = 0.326). Most AKI cases occurred within 72 h of LT (Figure 1a). Stage 3 AKI was more frequently observed in the EAD group than in the non-EAD group (*p* = 0.001; Figure 1b). Moreover, the incidence of AKD was higher in recipients with EAD than in those without it (*p* = 0.001; Figure 1a).

### 3.3. EAD and KRT Requirement Following LT

KRT was performed within 7 days of LT in 11 (15.3%) recipients in the non-EAD group and 14 (70.0%) recipients in the EAD group (*p* < 0.001). Six (6.5%) patients died within 7 days; all were in the EAD group and had received KRT prior to death. The cause of death in all six patients was profound hepatic failure. KRT treatment was maintained for ≥7 days in 7 (9.7%) in the non-EAD group and 7 (46.5%) in the EAD group (*p* = 0.002), respectively. The OR of EAD for KRT performance was estimated (Table 2), and EAD was an independent risk factor for both KRT requirement within 7 days after LT (*p* < 0.001) and prolonged KRT use for ≥7 days (*p* = 0.03).

**Figure 2:**
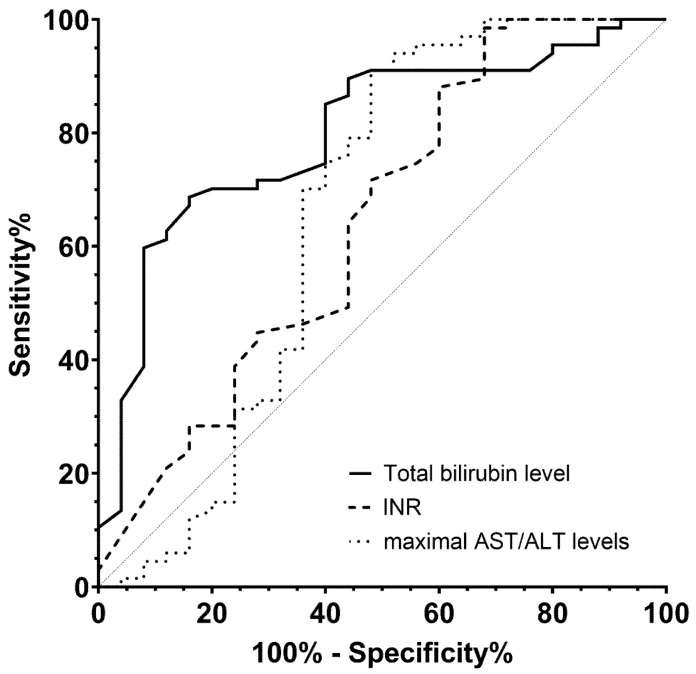
Prediction of KRT probability according to the EAD criterion. ROC curves were generated for each EAD component, including total bilirubin level, INR, and the maximal AST or ALT levels following LT. All components predicted the requirement for KRT within 7 days after LT: AUC of total bilirubin was 0.799 (95% CI 0.699, 0.899; *p* < 0.001); AUC of INR, 0.643 (95% CI 0.505, 0.781; *p* = 0.036); and, AUC of maximal AST/ALT, 0.657 (95% CI 0.501, 0.814; *p* = 0.021). KRT, kidney replacement therapy; EAD, early allograft dysfunction; ROC, receiver operating characteristic; AUC, area under the curve; CI, confidence interval; INR, international normalized ratio; AST, aspartate aminotransferase; ALT, alanine aminotransferase; LT, liver transplantation.

We further examined the ROC curve of each EAD criterion to predict KRT requirement (Figure 2). All components of EAD predicted the need for KRT within 7 days after LT (*p* for total bilirubin level < 0.001, *p* for INR = 0.036, and *p* for maximal AST or ALT level = 0.021).

### 3.4. Long-Term Kidney Function After LT According to the EAD Status

Among the included participants, kidney function 3 months after LT was evaluated in 83 recipients, excluding 7 who died (6 due to hepatic failure and 1 due to a biliary complication), 1 with graft failure, and 1 who was followed up for <3 months. The baseline eGFR was 107.4 (92.8, 115.6) mL/min/1.73 m^2^ and 100.8 (83.6, 110.1) mL/min/1.73 m^2^ in recipients without EAD and with EAD, respectively (*p* = 0.355), whereas their 3-month eGFR was 103.4 (91.0, 113.8) mL/min/1.73 m^2^ and 100.7 (95.1, 106.5) mL/min/1.73 m^2^, respectively (*p* = 0.457).

**Figure 3:**
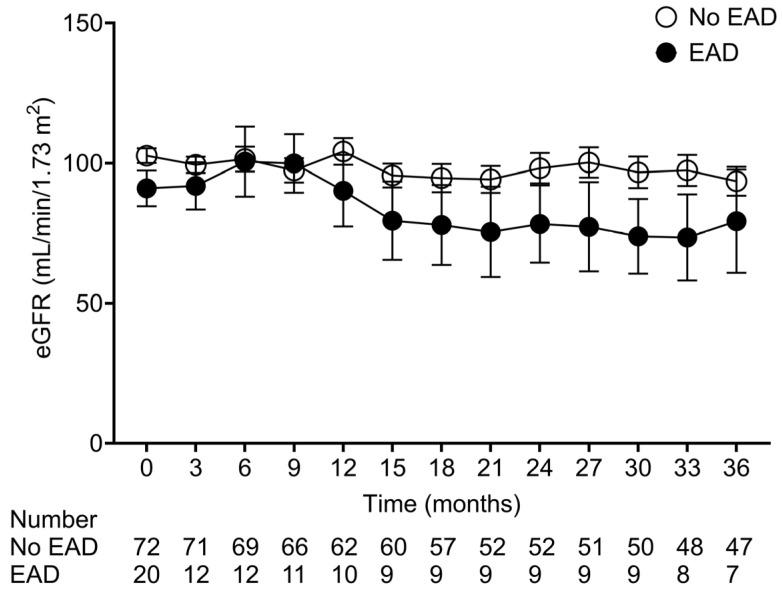
The eGFR pattern after LT based on the EAD status. After 3 months following LT, the eGFR in the non-EAD group decreased by −2.2 mL/min/1.73 m^2^ (95% CI −2.8, −1.6) per year (*p* < 0.001). Despite the statistical insignificance, the eGFR in the EAD group exhibited a steeper decline by −3.4 mL/min/1.73 m^2^ (95% CI −4.8, −1.9) per year compared to the non-EAD group (*p* = 0.090). LT, liver transplantation; EAD, early allograft dysfunction; eGFR, estimated glomerular filtration rate; CI, confidence interval.

The eGFR trajectories were explored pre- and post-LT based on the EAD status (Figure 3). The 3-month eGFR was comparable between the groups (*p* = 0.614); however, a trend toward a steeper decline in eGFR was observed in recipients in the EAD group (*p* = 0.090). During the entire follow-up period, 23 deaths occurred. Although the mortality rate was numerically higher in the EAD group than in the non-EAD group (40.0% vs. 20.8%), the difference in overall survival was not statistically significant (*p* = 0.468).

### 3.5. Hospitalization and Calcineurin Inhibitor Exposure

**Figure 4:**
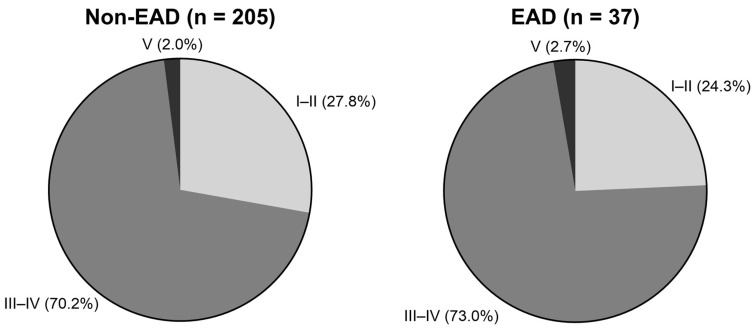
The severity of hospitalization after LT discharge by EAD status. The distribution of severity was assessed using the Clavien–Dindo grades among hospitalization episodes within 1 year after LT. In the non-EAD group, 27.8% of episodes were grades I–II; 70.2%, grades III–IV; and 2.0%, grade V. On the other hand, 24.3% were grades I–II; 73.0%, grades III–IV; and 2.7%, grade V. LT, liver transplantation; EAD, early allograft dysfunction.

To determine the factors other than EAD influencing kidney function, we first reviewed hospitalization events among recipients discharged after LT admission. The number of hospitalizations was 5 (2, 9) and 3 (1, 10) in the non-EAD and EAD groups, respectively (*p* = 0.472). In the negative binomial regression with an offset for follow-up duration, EAD was not associated with the hospitalization rate (incidence rate ratio, 1.13; *p* = 0.726). Thereafter, we evaluated the severity of hospitalization within 1 year after LT based on the Clavien–Dindo classification system (Figure 4), and the distribution was comparable regardless of the EAD status *p* = 0.879).

In addition, we examined calcineurin inhibitor exposure in both EAD groups. A renal-sparing intervention (minimizing tacrolimus and introducing everolimus) was applied in 19 recipients during follow-up, including 5 (25.0%) in the EAD group and 14 (19.4%) in the non-EAD group (*p* = 0.550). The trough levels of tacrolimus were obtained, and normalized values were estimated after calculating the AUC of tacrolimus exposure. The normalized trough levels of tacrolimus in the two groups were comparable, i.e., 6.2 (5.6, 7.0) ng/mL in the non-EAD group and 5.9 (4.7, 6.5) ng/mL in the EAD group (*p* = 0.189). Chronic rejection occurred in 4 recipients during follow-up, all in the non-EAD group, and was associated with medication non-compliance.

## 4. Discussion

This study aimed to investigate the clinical impact of EAD on post-LT kidney outcomes. Recipients with EAD had a higher risk of developing severe AKI and AKD, and they required KRT more frequently and for a longer duration after LT than those without EAD. All EAD components, including total bilirubin level, INR, and maximal AST/ALT levels, were associated with KRT performance following LT. In addition, recipients with EAD seemed to exhibit steeper declines in eGFR than those without EAD, even though hospitalization burden and calcineurin inhibitor exposure were similar regardless of EAD status.

Postoperative AKI is a critical complication that increases the short- and long-term morbidity and mortality [15]. It directly or indirectly contributes to a cascade of adverse outcomes, including a higher risk of death, greater occurrence of postoperative complications, longer hospital stay, development of CKD, and increases in healthcare and societal burdens [18,19]. In this context, AKI can be considered a sentinel event in postoperative management. Both AKI and CKD are common after LT, with reported incidence rates ranging 52–80% and 10–45%, respectively. Their clinical implications, such as prolonged hospitalization, accelerated CKD progression, and increased patient mortality, have been reported [4,5,6,7,8,9,10]. Managing modifiable risk factors for renal dysfunction is crucial for preserving perioperative kidney function. Among these, EAD is of particular interest. This study focused on EAD to assess its impact on adverse kidney outcomes after LT. Wadei et al. [11] demonstrated that EAD is a risk factor for both AKI and CKD requiring KRT in LT recipients. Nevertheless, the overall spectrum of kidney complications resulting from EAD has not been fully elucidated, and the mechanisms underlying this relationship remain unclear. In this study, we aimed to comprehensively evaluate the effect of EAD on both short- and long-term kidney dysfunction and explore the potential factors contributing to its adverse effects.

This study first evaluated the incidence and severity of AKI according to the EAD status. Compared to recipients without EAD, recipients with EAD experienced markedly higher rates of severe AKI and a greater need for KRT within the first 7 postoperative days after LT. Notably, most AKI events occurred within the first 2 days post-LT, suggesting that patient-, surgery-, and anesthetic-related factors, such as pre-existing health conditions, emergency operation, and perioperative hemodynamic instability, play a major role in the development of AKI [19]. Although not statistically significant, early onset AKI was prominent in patients who were subsequently classified into the EAD group, indicating a close mechanistic link between the two conditions. Given the emerging evidence, EAD as a consequence of hepatic ischemia–reperfusion injury induces oxidative stress and systemic inflammation, which has been reported as the mechanism for the development of AKI [20,21,22]. In contrast, prolonged hepatic dysfunction can contribute to AKI through the mechanism of cirrhosis-induced AKI, such as hemodynamic instability, cirrhotic cardiomyopathy, systemic inflammation, bacterial translocation, and bile acid toxicity [12]. This mechanistic continuum may explain the frequent coexistence of EAD and AKI in critically ill LT recipients. In this study, older donor age and prolonged cold ischemia time were associated with EAD, both of which are recognized contributors to hepatic ischemia–reperfusion injury. In this context, efforts to reduce such injury, including the use of machine perfusion, may help prevent EAD and mitigate post-LT kidney complications. Furthermore, the development of biomarkers and an updated consensus definition of EAD are warranted for its recognition and management after LT.

This study further evaluated the role of each EAD component in predicting the KRT need within 7 days after LT. Although all EAD parameters were associated with postoperative KRT requirement, the total bilirubin levels ≥ 10 mg/dL on postoperative day 7 appeared to be the strongest predictor of AKI necessitating KRT. Serum AST and ALT levels primarily reflect hepatocellular injury, whereas total bilirubin level and INR represent hepatic excretory and synthetic functions, respectively [23]. Thus, our results indicate that delayed allograft function is mainly responsible for renal impairment requiring KRT, rather than for new or ongoing hepatic or kidney damage. In contrast, bile acid toxicity can be considered as another plausible mechanism linking severe cholestasis to kidney injury [24,25]. Therefore, sustained hyperbilirubinemia can be considered an important biomarker of post-LT kidney dysfunction, warranting an early multidisciplinary approach involving hepatobiliary surgeons and nephrologists for LT recipients with severe cholestasis.

We evaluated the duration of kidney dysfunction according to EAD status and found that AKD was more common among recipients with EAD than among those without EAD. Moreover, EAD was independently associated with prolonged KRT use (≥7 days). In addition to severity, duration and reversibility of injury are important determinants of both short- and long-term outcomes [26,27]. This was also confirmed in a study by Chiu et al. [10], who reported that LT recipients with recovered AKI had a comparable risk of CKD development to those without AKI, whereas patients with unrecovered AKI had a higher risk. In summary, EAD is associated with severe and prolonged kidney dysfunction, which can predispose to have CKD. These findings suggest that proactive measures to support renal recovery are warranted to preserve long-term kidney function, particularly in LT recipients with EAD.

A previous study reported an association between EAD and end-stage kidney disease requiring KRT [11]. However, whether this risk depends on the accelerated rate of CKD progression or is related to severe and frequent events that are prone to necessitate KRT is unclear. To explain this finding, we examined the long-term renal function trajectory in the EAD group. At baseline and 3-month post-LT, the eGFR did not differ regardless of EAD status; in contrast, eGFR showed a steeper decline over time in recipients who experienced EAD. Although this difference was not statistically significant, the results suggested that the negative impact of EAD may persist beyond the immediate postoperative period. This finding underscores the importance of continued long-term renal monitoring in patients with EAD. From a pathophysiological perspective, the steeper decline in the eGFR in recipients with EAD may be caused by various nephrotoxic insults other than EAD. Therefore, we explored hospitalization burden and calcineurin inhibitor exposure as known nephrotoxic insults. Based on our results, EAD was not associated with the risk of hospitalization and was unrelated to the severity of hospitalization within 1 year following LT. This contradicts the hypothesis that the negative impact of EAD on long-term kidney function is responsible for EAD-associated frequent and severe complications following LT. In contrast, the toxicity of calcineurin inhibitors might not be the reason for EAD-associated CKD progression, given that calcineurin inhibitor exposure was similar between the EAD groups. These findings are noteworthy because they suggest that the worse kidney outcomes observed in EAD were not simply a byproduct of patients with EAD experiencing more general illnesses or receiving more nephrotoxic immunosuppression. In other words, EAD may be a determinant of both short- and long-term renal dysfunction. These findings can be explained by the AKI–CKD continuum [19]; however, further studies are essential to identify the pathophysiological details, considering other confounding factors.

This study has several limitations. First, this study was analyzed with a small sample size. This can restrict the power to detect certain differences; thus, it increases the susceptibility to type II errors. The small sample size also limited the number of covariates used for adjustment and resulted in a wide range of confidence intervals, which might have contributed to the bias and limited the interpretation. Furthermore, the number of evaluable patients decreased substantially during follow-up, particularly in the EAD group; therefore, the long-term eGFR trajectory should be interpreted with caution, as it was based on a relatively small subset of surviving patients and may have been affected by survivor bias. Second, the definition of EAD used was the most widely validated one; however, its binary nature lacks granularity and fails to capture the continuum of graft dysfunction compared to other scoring tools [28,29]. Nevertheless, our definition could allow the assessment of the individual impacts of EAD components on KRT requirement. Third, we did not measure certain granular factors such as hemodynamic data, including intraoperative blood pressure or kidney injury biomarkers, which could provide further insight into the mechanisms linking EAD with AKI. Finally, the utility of serum creatinine levels in LT recipients should be examined. Serum creatinine levels are known to overestimate renal function in patients with advanced liver disease [30]. Serum cystatin C levels could offer a more accurate assessment in patients before [31] as well as after LT.

## 5. Conclusions

This study investigated the relationship between EAD and kidney outcomes in LT recipients and found that EAD could be an independent risk factor for overall adverse kidney outcomes such as severe AKI and AKD and accelerated CKD progression in LT recipients. Therefore, patients who develop EAD should be monitored closely for renal complications, and proactive management is essential to support renal function following LT. Further studies with a larger sample size are required to determine whether strategies for preventing EAD can help improve kidney outcomes as well as allograft and patient outcomes in these vulnerable patients.

## Figures and Tables

**Table 1:** Characteristics of LT recipients according to the early allograft dysfunction status.

Variable	No EAD (n = 72)	With EAD (n = 20)	*p*
*Recipient factors*			
Age	55 (48, 61)	54 (52, 61)	0.970
Male	50 (69.4)	13 (65.0)	0.915
Body mass index	23.8 (20.6, 25.8)	24.0 (21.5, 26.5)	0.523
Diabetes	22 (30.6)	6 (30.0)	1.0
Hypertension	15 (20.8)	4 (20.0)	1.0
Cause of liver disease			0.014
Malignancy	20 (27.8)	1 (5.0)	
Hepatitis B virus	10 (13.9)	5 (25.0)	
Hepatitis C virus	3 (4.2)	0 (0)	
Alcoholic liver disease	31 (43.1)	9 (45.0)	
Autoimmune or others	5 (6.9)	0 (0)	
Acute fulminant hepatitis	3 (4.2)	5 (25.0)	
Laboratory			
Aspartate transferase	49 (34, 72)	47 (30, 73)	0.708
Alanine transferase	21 (15, 38)	31 (19, 88)	0.154
Alkaline phosphatase	129 (96, 162)	123 (96, 175)	0.726
Total bilirubin	2.2 (1.4, 8.8)	4.6 (1.2, 22.0)	0.636
Albumin	3.0 (2.7, 3.6)	3.3 (3.0, 3.6)	0.353
International normalized ratio	1.5 (1.2, 2.1)	1.8 (1.3, 2.5)	0.349
Platelet count	75 (52, 126)	68 (37, 127)	0.457
Sodium	137 (134, 139)	139 (136, 140)	0.049
Creatinine	0.8 (0.6, 1.2)	1.5 (0.9, 4.0)	0.021
Baseline kidney function			
Creatinine	0.6 (0.6, 0.8)	0.8 (0.6, 1.0)	0.084
eGFR	107.3 (92.4, 115.6)	98.2 (79.0, 110.1)	0.116
MELD score	15 (10, 29)	30 (13, 40)	0.049
KRT before LT	8 (11.1)	7 (35.0)	0.017
*Donor factors*			
Deceased donor	21 (29.2)	10 (50.0)	0.140
ABO incompatibility	8 (11.1)	2 (10.0)	1.0
Donor age	42 (28, 48)	48 (36, 65)	0.020
Male donor	44 (61.1)	14 (70.0)	0.641
Cold ischemia time	1.3 (1.0, 3.1)	2.9 (1.1, 5.4)	0.048
Warm ischemia time	36 (30, 42)	40 (32, 46)	0.203
Graft steatosis	6 (8.3)	2 (10.0)	1.0
GRWR < 0.8	8 (11.1)	2 (10.0)	1.0
*Operative factors*			
Operative time	478 (419, 536)	460 (400, 562)	0.922
Estimated blood loss	5000 (3000, 9250)	10,000 (3750, 17,000)	0.139
Transfusion	1900 (1080, 3105)	3600 (1620, 4895)	0.056

Data are expressed as median (interquartile range) or number (percentage). The *p* value for cause of liver disease represents the overall comparison of the distribution of etiologies between the groups using the chi-square test. The creatinine value under “Laboratory” indicates the immediate pre-transplant serum creatinine, whereas the value under “Baseline kidney function” represents the nadir serum creatinine measured within 12 months before transplantation during a clinically stable period. EAD, early allograft dysfunction; eGFR, estimated glomerular filtration rate; MELD, Model for End-Stage Liver Disease; KRT, kidney replacement therapy; GRWR, graft-to-recipient weight ratio.

**Table 2:** Association between clinical variables and KRT requirement following liver transplantation.

Variable	KRT Within 7 Days				Prolonged (≥7 Days) KRT			
	Univariate OR (95% CI)	*p*	Multivariate OR (95% CI)	*p*	Univariate OR (95% CI)	*p*	Multivariate OR (95% CI)	*p*
Age	0.9 (0.9–1.0)	0.011	1.0 (0.9–1.0)	0.280	0.9 (0.9–1.0)	0.007	1.0 (0.9–1.1)	0.607
Male	1.0 (0.4–2.7)	0.952			0.8 (0.3–3.0)	0.758		
Acute hepatitis	10.3 (2.2–74.1)	0.007	0.5 (0.0–5.7)	0.568	1.1 (0.1–7.2)	0.968		
Baseline Cr	10.6 (2.6–70.9)	0.005	12.0 (1.3–143.3)	0.027	3.0 (0.9–10.8)	0.068	1.5 (0.4–7.2)	0.534
MELD	1.2 (1.1–1.2)	<0.001	1.3 (1.1–1.5)	<0.001	1.2 (1.1–1.3)	<0.001	1.1 (1.0–1.2)	0.002
Donor age	1.1 (1.0–1.1)	<0.001	1.0 (0.9–1.0)	0.253	1.1 (1.0–1.2)	0.001	1.0 (1.0–1.1)	0.540
Male donor	0.5 (0.2–1.4)	0.183			0.7 (0.2–2.2)	0.474		
GRWR < 0.8	1.9 (0.5–7.5)	0.340			3.0 (0.6–13.5)	0.152		
Cold ischemia time ≥ 6 h	3.1 (0.8–12.2)	0.098	0.1 (0.0–0.8)	0.030	0.6 (0.0–3.9)	0.670		
Warm ischemia time ≥ 50 min	1.6 (0.5–5.3)	0.438			0.4 (0.0–2.5)	0.442		
EAD	12.9 (4.3–43.9)	<0.001	55.7 (5.4–1677.6)	<0.001	8.1 (2.3–30.4)	0.001	6.3 (1.3–41.4)	0.025

Multivariate analysis was adjusted for variables with *p* < 0.1 in the univariate analysis. CI, confidence interval; EAD, early allograft dysfunction; GRWR, graft-to-recipient weight ratio; KRT, kidney replacement therapy; MELD, Model for End-stage Liver Disease.

## Data Availability

Data supporting the findings of this study are available from the corresponding author upon request.
